# Regulating Light Absorption, Charge Orientation, and Oxygen Activation of Conjugated Porous Polymers for Photocatalytic Organic Synthesis

**DOI:** 10.1021/accountsmr.5c00230

**Published:** 2025-10-24

**Authors:** Zhu Gao, Sizhe Li, Juntao Tang, Jiayin Yuan, Guipeng Yu

**Affiliations:** † Hunan Key Laboratory of Micro & Nano Materials Interface Science, College of Chemistry and Chemical Engineering, 12570 Central South University, Lushan South Road 932, Changsha 410083, Hunan, P. R. China; ‡ School of Chemistry, 56711 Southwest Jiaotong University, Chengdu, Sichuan 610031, P. R. China; § College of Smart Materials and Future Energy, 12478 Fudan University, 200433 Shanghai, P. R. China; ∥ Department of Chemistry, 7675 Stockholm University, Svante Arrhenius väg 16 C, 106 91 Stockholm, Sweden

## Abstract

Conjugated porous polymers (CPPs), featuring π-conjugation systems, freedom in molecular structural design, and intrinsic porosity, have emerged as a modular platform for visible-light-driven organic synthesis. At present, their photocatalytic efficiency is limited by incomplete absorption of visible light, inefficient charge separation, and inadequate management of oxygen-active species, urging the field to explore solutions. Light absorption can be strengthened by molecular engineering strategies, e.g., extension of π-conjugation, adjustment of donor–acceptor units, and incorporation of chromophores, e.g., triazine and phenothiazine, that redshift and thus broaden the absorption. Charge separation can intensify by integration of donor–acceptor segments and π-bridged linkers to cut exciton binding energy and extend lifetime of carriers; migration of charge carriers can be more directed by introduction of polar substituents and localized dipoles. Along with modifying the bandgap structure, modulation of the catalytic microenvironment can shape selective substrate activation, for instance, framework rigidification, control of electronic structure of active sites, and spatial confinement of intermediates. In terms of handling oxygen-active species, we can regulate charge distribution and electronic structure within the conjugated backbone. This regulation enhances formation of reactive intermediates such as superoxide, hydroxyl radical, and other essential oxygen-derived species to drive oxidative photocatalytic processes. Together, these approaches establish a coherent design scheme to develop high-performance, metal-free photocatalysts for diverse organic synthesis and sets a foundation for future sustainable catalysis and synthesis of photoresponsive materials.

## Graphical abstract

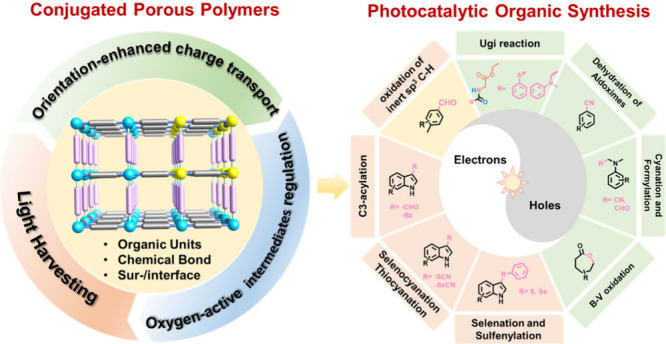

## 1 Introduction

Photocatalysis has attracted increasing attention in organic synthesis due to potential bond-forming reactions under mild conditions or molecules inaccessible via other means.^1–3^ Organocatalysis driven by visible light, despite widespread interest due to its energy effectiveness, remains constrained by several interlocked limitations that originate from fundamental photophysical and redox aspects. A significant proportion of photocatalytic systems are limited in their ability to absorb visible light, which suppresses excitation efficiency and in turn photon utilization. Even when excitation occurs, short exciton lifetimes and the inefficient separation of charge carriers cause high rates of recombination and low redox efficiency. On top of that, the uncontrolled formation, accumulation, and transformation of oxygen-active species often result in nonselective pathways and impede desirable oxidative conversions.^4–6^ These issues are inherently coupled and hard to address individually, which requires a solution based on molecular design.

Conjugated porous polymers (CPPs) contain π-conjugated networks in a cross-linked porous form. They usually include conjugated microporous polymers (CMPs), porous aromatic frameworks (PAFs), and covalent organic frameworks (COFs). CPPs, either amorphous or crystalline in nature, are important heterogeneous photocatalysts,^7–9^ benefiting from massive structural tunability by organic reactions and sufficient chemical and thermal stability for organic synthesis due to high cross-linking densities. CPPs offer noticeably advantages over traditional inorganic semiconductors, in particular in connection to precise modulation of optical and electronic properties through judicious choice of monomers and targeted postsynthetic modifications. These characteristics position CPPs as a unique platform for light-driven organic synthesis.^10–13^ Despite bright prospects, their photocatalytic performance is persistently limited by structural constraints related to light harvesting, charge dynamics, and oxygen-active species. Many CPPs fail to absorb visible light sufficiently due to imperfect conjugation or mismatched energy levels between donor and acceptor units. Even under successful photoexcitation, the inadequate internal polarity and/or dipole orientation deteriorates separation and migration of charge carriers, resulting in rapid recombination. At the catalytic interface, the absence of a well-defined microenvironment compromises the formation of oxygen-active species and impairs oxidation selectivity and redox balance. These limitations have a molecular origin and arise from imperfect positions of functional electronic motifs along the polymer backbone, affecting the extent of conjugation, the distribution of donor–acceptor units, and the internal polarity. Addressing them requires precise molecular manipulation of connectivity and electronic structure of repeating units, thus simultaneously handling light absorption, charge transport, and catalytic reactivity.

This Account discusses the molecular structure design of CPPs to dictate key processes in photocatalytic organic synthesis. Our efforts cover three key aspects, i.e., enhancing visible-light absorption via conjugation extension and bandgap modulation, driving directional charge separation via donor–acceptor alignment and internal polarity, and optimizing local electronic microenvironment at reactive sites to dictate oxygen-active species. These efforts guide us successfully to CPP-based systems achieving efficient and selective photocatalytic synthesis of organic molecules under visible light.

## 2 Overview of Photocatalysis of CPPs

CPPs are formed through either irreversible or reversible covalent coupling of different organic building blocks.^14–16^ Their modular structure, permanent porosity, and adequate stability under thermal and chemical conditions make them suitable for systematic tuning of the optoelectronic properties at the molecular level. Unlike conventional inorganic semiconductors, CPPs can consist entirely of light elements and are metal-free with adjustable conjugation, donor–acceptor arrangement, and polarity. These characteristics offer opportunities to address long-standing challenges in photocatalytic organic synthesis mentioned above.^17–22^

CMPs,^23–25^ CTFs,^26–28^ and π-conjugated PAFs^29,30^ represent key structural classes within the broad family of CPPs. Depending on the synthetic routes, they can exist in an amorphous or crystalline form.^31–35^ Through the deliberate choice of building blocks and coupling motifs and the involved organic synthetic tools, these systems allow for extension of π-conjugation, alignment of donor–acceptor electronic units, and configuration of the local polarity. Such molecular-level engineering, if played well, can simultaneously manage light absorption characteristics, spatial separation, and transport of photoinduced charges and generation, diffusion, and reactivity of oxygen species involved in photocatalytic steps.

### Light Absorption

Photoexcitation in organic semiconductors typically generates excitons with diffusion lengths on the length scale of tens of nanometers. Effective dissociation into free carriers requires adequate energy offsets between adjacent donor and acceptor pairs as well as internal electric fields established by asymmetric charge distribution. In CPPs of well-designed chemical structures, such features can be embedded through the spatial organization of polar linkers or gradients in local electronic environments. These factors advance charge separation, suppress recombination, and extend carrier lifetimes.

### Exciton Dissociation and Charge Separation

Following photon absorption, the generation of excitons must be accompanied by efficient charge separation to avoid recombination losses. In well-designed CPPs, donor–acceptor motifs and internal electric fields drive directional migration of photogenerated charge carriers. π-Bridged linkers and polarity gradients within the framework further facilitate charge delocalization, prolonging the lifetime of oxygen-active species. These molecular features directly correlate with the photocatalytic performance across diverse reaction systems.

### Surface Redox Activity

Upon charge separation, electrons in the lowest unoccupied molecular orbital (LUMO), which correspond to the conduction band minimum, are involved in reduction steps, while holes in the highest occupied molecular orbital (HOMO), aligned with the valence band maximum, participate in oxidation processes. The effectiveness of these redox events is determined by the correspondence between the energy levels of the polymer and the redox potentials of the reacting species. In addition to thermodynamic compatibility, the local environment of the active sites, including pore confinement and electronic polarity, impacts the formation and transformation of oxygen-related intermediates that mediate key photocatalytic reactions.

**1:**
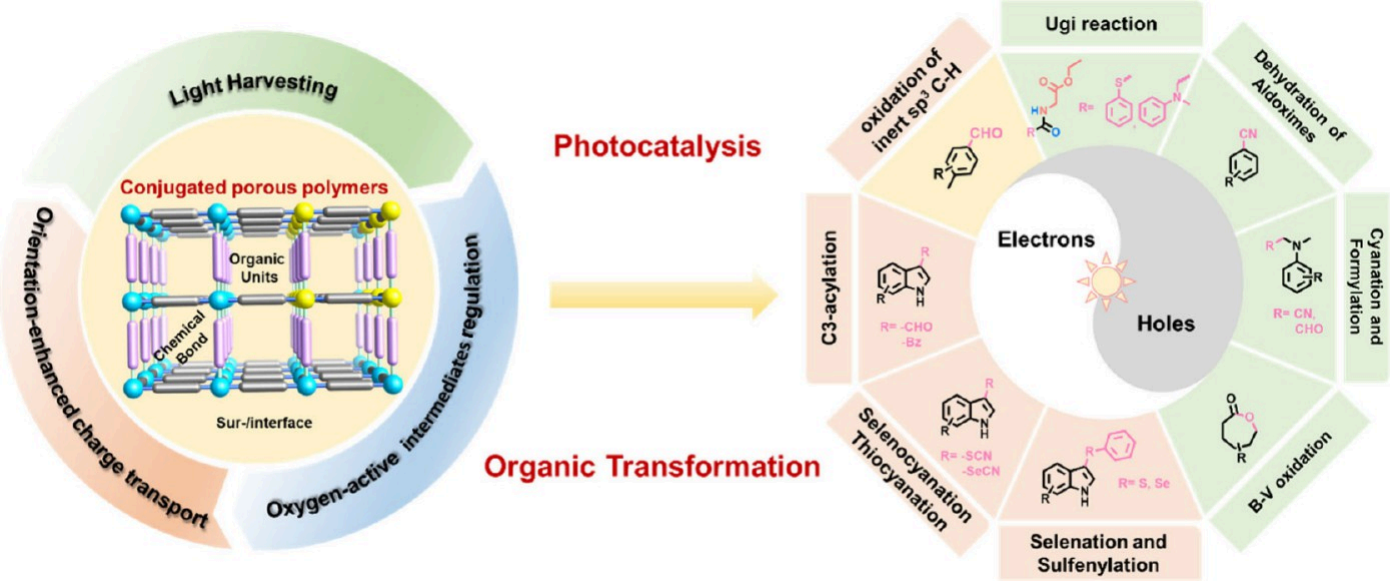
Schematic Illustration of Functionalized CPPs for Photocatalytic Organic Synthesis and Overview of the Topics Covered in This Account

Next, we discuss representative molecular design strategies applied by us to address each of these aspects and examine their roles in determining the reactivity and selectivity, sharing our experience in visible-light-driven organic synthesis.

## 3 Improving Light Harvesting in CPPs

Efficient light absorption is essential for operating photocatalytic processes in CPPs, as it governs the generation of excited states and the availability of charge carriers for subsequent redox reactions. To improve solar light harvesting, our molecular design efforts focus on extending π-conjugation, incorporating photosensitizers, tuning the bandgap through copolymerization, and functionalizing the backbone with tailored substituents.

**Figure 1:**
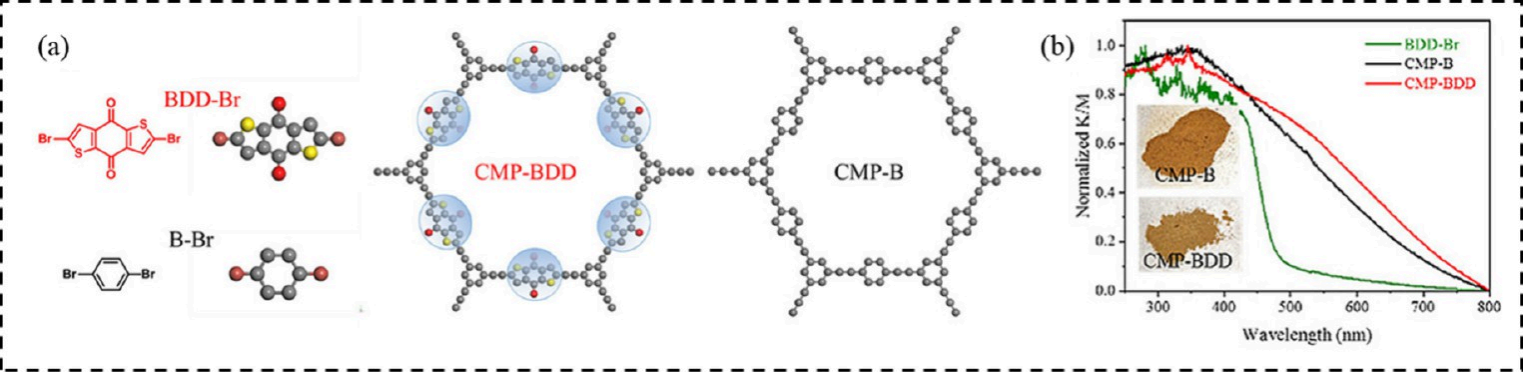
(a) Chemical structures of synthesized monomers (BDD-Br and B-Br) and resulting conjugated microporous polymers (CMP-BDD and CMP-B); (b) UV–vis diffuse reflectance spectra (DRS) of BDD-Br and CMPs. Adapted with permission from ref 36. Copyright 2019 Wiley-VCH.

### 3.1 Extending π-Conjugation

Modulation of the π-conjugated framework is a foundational strategy for raising the light-harvesting properties of CPPs. Extension of π-delocalization narrows down the optical bandgap and thereby absorbs lower-energy photons across the visible spectrum. Our representative case involves the incorporation of benzo­[1,2-b:4,5-b′]­dithiophene-4,8-dione (BDD) into CMPs (Figure 1). The resulting CMP-BDD was synthesized via Sonogashira–Hagihara coupling between BDD and 1,3,5-triethynylbenzene, yielding a porous network with promoted π-conjugation and strong electron-deficient character.^36^ Diffuse reflectance UV–vis spectroscopy showed that CMP-BDD exhibits a broad absorption band centered at 500 nm, substantially red-shifted relative to that of the benzene core. In comparison with a structurally analogous CMP lacking the BDD moiety (CMP-B), CMP-BDD demonstrated markedly enhanced absorption in the visible region, which is attributable to the extended conjugation introduced by the BDD units. Kubelka–Munk-transformed spectra indicated that the optical bandgap of CMP-BDD was 2.06 eV, narrower than the 2.25 eV of CMP-B. Electrochemical analysis further revealed a lowered LUMO energy level in CMP-BDD, thereby increasing the thermodynamic driving force for photoinduced electron transfer. The integration of BDD motifs, characterized by rigid planarity and strong electron-withdrawing capability, promotes π-delocalization and orbital-level reconfiguration, which collectively enhance visible-light absorption and facilitate bandgap reduction.

### 3.2 Incorporating Photosensitizers

Incorporating photosensitive units into CPPs can expand the light absorption in the visible region. Among various candidates, ferrocene stands out for its electron-rich metallocene core, reversible redox behavior, and capacity to mediate intramolecular charge transfer, making it particularly suitable for light-harvesting applications. The incorporation of ferrocene units into CPPs extends the π-conjugated backbone, creating additional photoactive sites that enhance light absorption and facilitate charge transfer.

**Figure 2:**
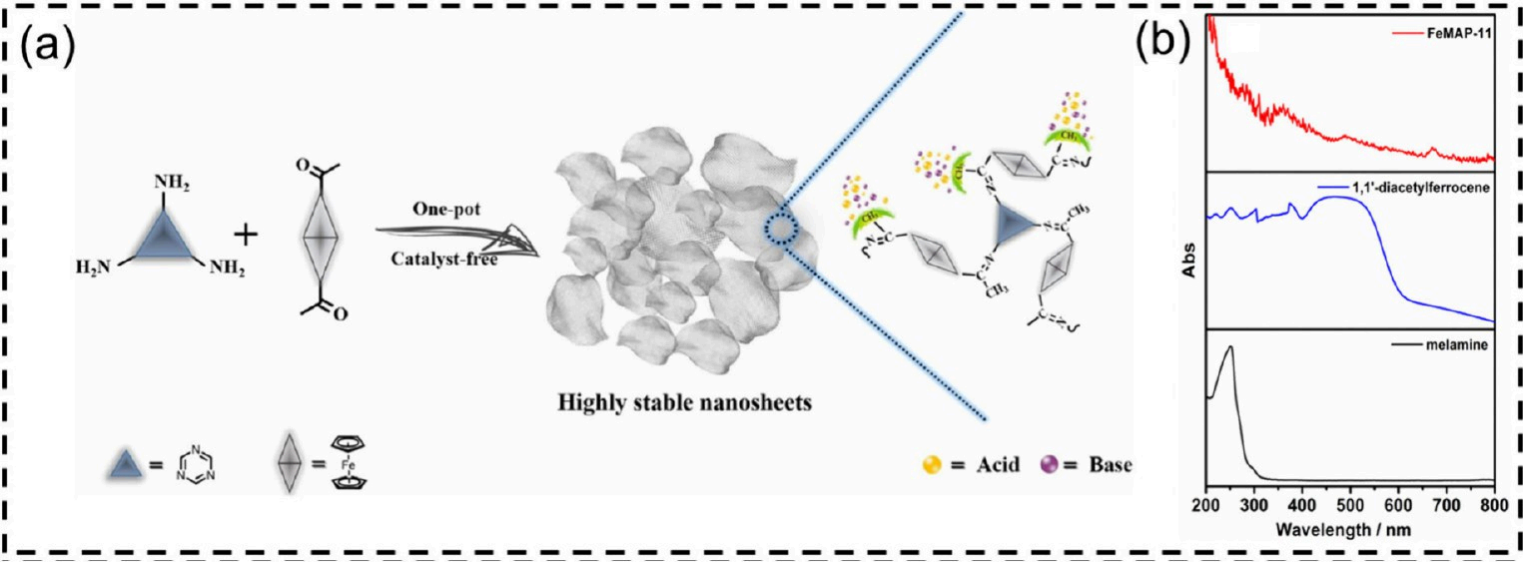
(a) Synthetic route to ferrocene-based microporous aromatic polymer nanosheets (FeMAP-11); (b) UV–vis spectra of FeMAP-11 (red), melamine (black), and diacetylferrocene (blue). Adapted with permission from ref 37. Copyright 2020 Elsevier.

Our study involves the synthesis of ferrocene-containing microporous aromatic polymer nanosheets (FeMAP-11) via polymerization of 1,1′-diacetyleneferrocene with 1,3,5-triethynylbenzene (Figure 2).^37^ The resulting framework integrates the redox-active FeCp_2_ moiety within a conjugated porous backbone. Solid-state UV–vis spectra revealed a broad absorption range of 300–600 nm, with a maximum at 365 nm. This enhanced absorption was attributed to π-delocalization across the ferrocene-bridged network, which was supported by fluorescence and diffuse reflectance measurements. Although FeMAP-11 presented high activity and recyclability in photocatalytic atom transfer radical polymerization (ATRP), its boosted light-harvesting ability was identified as the primary contributor to its photocatalytic performance. Such integration helps in the development of metal–organic hybrid CPPs with tunable optical responses and enhanced photocatalytic activity.

### 3.3 Bandgap Tuning via Copolymerization

Beyond the introduction of photosensitizing units, adjusting their incorporation levels within polymeric frameworks provides additional control over light absorption. In copolymer systems, the band structure can be tuned by varying the electronic properties and ratios of monomers, enabling flexible control of visible-light absorption.

In 2023, we constructed phenothiazine (PTH)-anchored covalent triazine frameworks (CTFs).^38^ Phenothiazine, a redox-active heterocycle with an extended π-system and strong electron-donating character, serves as both a visible-light-absorbing chromophore and a charge-transfer mediator (Figure 3). Monofunctionalized PTH was copolymerized with a triazine-phenyl backbone, forming a series of crystalline frameworks denoted as CTF-PTH-*x*, where *x* refers to the molar percentage of PTH units (1%, 3%, and 5%). The pristine CTF 1 absorbed mainly in the UV region (<400 nm), while adding the PTH content extended absorption into the visible region up to 750 nm, a shift attributed to expansion of π-conjugation and stronger donor–acceptor interactions. This copolymerization approach is simple and betters charge separation as well, with electrons localized on triazine units to activate molecular oxygen and generate oxygen-active species, while holes on the sulfur atoms of PTH oxidized substrates. These complementary pathways efficiently photocatalyzed oxidative reactions, e.g., in benzimidazole synthesis, oxidative formylation, and aldoxime dehydration.

### 3.4 Functionalization with Tailored Functional Groups

Incorporating electron-withdrawing substituents into the CPP backbone can tune its electronic structure to narrow down the bandgap, improve charge separation, and enhance visible-light absorption. We applied this strategy to phenothiazine-based CMPs, where fluorine substituents of different content were introduced (Figure 4).^39^ The high electronegativity of fluorine generated local dipolar fields to drive charge delocalization and carrier separation, as confirmed by spectroscopic analysis and density functional theory calculations. UV–vis diffuse reflectance spectroscopy revealed an absorption threshold up to 500 nm and a bandgap reduction from 2.56 eV in the unsubstituted polymer to 2.42 eV in its fluorinated analogue. These electronic enhancements translated into higher photocatalytic activity, with the fluorinated materials achieving efficient oxidative cyanation of tertiary amines to α-aminonitriles under mild, metal-free conditions. Improved light harvesting and charge separation also promoted the formation of oxygen-active species under visible light, contributing to oxidative transformation.

## 4 Orientation-Enhanced Charge Transport

CPP-based materials, benefiting from the structural diversity of organic linkers and synthetic versatility, offer unique microenvironments around catalytic sites and allow for alternative reaction pathways distinct from those in homogeneous solution. In photocatalytic redox processes, charge separation and transfer are critical because photogenerated charges require hundreds of picoseconds to migrate from the bulk to reactive sites, whereas bulk recombination can occur within only a few picoseconds. Strategies have been developed to promote directional charge transfer in CPP-based photocatalysis, thereby improving the utilization of photogenerated carriers.

### 4.1 D-A Effect

Constructing donor–acceptor (D-A) systems can accelerate charge separation and transfer in CPP-based photocatalysis, as the electron push–pull interaction between donor and acceptor units speeds up carrier migration. Optimizing the acceptor motifs further strengthens this effect, as demonstrated by carbazole-based polymers that rapidly transfer electrons from the donor to acceptor segments. To this end, we synthesized a series of carbazole-containing CPPs (CMP-CSU5, CMP-CSU6, and CMP-CSU7) via FeCl_3_-mediated Friedel–Crafts reactions,^40^ with redox potentials tuned by varying the acceptor motifs (1,4-dibenzyl, 1,3,5-tribenzyl, and 1,3,5-triazin-2,4,6-triyl) (Figure 5).

**Figure 3:**
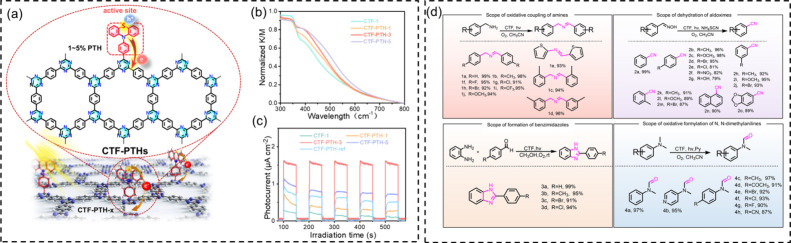
(a) Molecular design for unidirectional electron transfer on phenothiazine sites in CTFs; (b) UV–vis diffuse reflectance spectra; (c) photocurrent response; (d) substrate scope for CTF-catalyzed oxidative coupling of primary amines, aldoxime dehydration, benzimidazole synthesis, and oxidative formylation of *N*,*N*-dimethylanilines. Adapted with permission from ref 38. Copyright 2023 American Chemical Society.

Exploiting CPPs to generate oxygen-active species under visible light (λ > 420 nm), we employed the C-3 formylation and thiocyanation of indoles to evaluate photocatalytic performance. Among them, CMP-CSU6, derived from 1,3,5-tri­(9*H*-carbazol-9-yl)­benzene and exhibiting the strongest oxidative capability, showed more efficient charge separation and transfer than CMP-CSU5 and CMP-CSU7, resulting in superior activity in both reactions. These results underscore the effectiveness of D-A modulation in improving the CPP photocatalytic performance.

**Figure 4:**
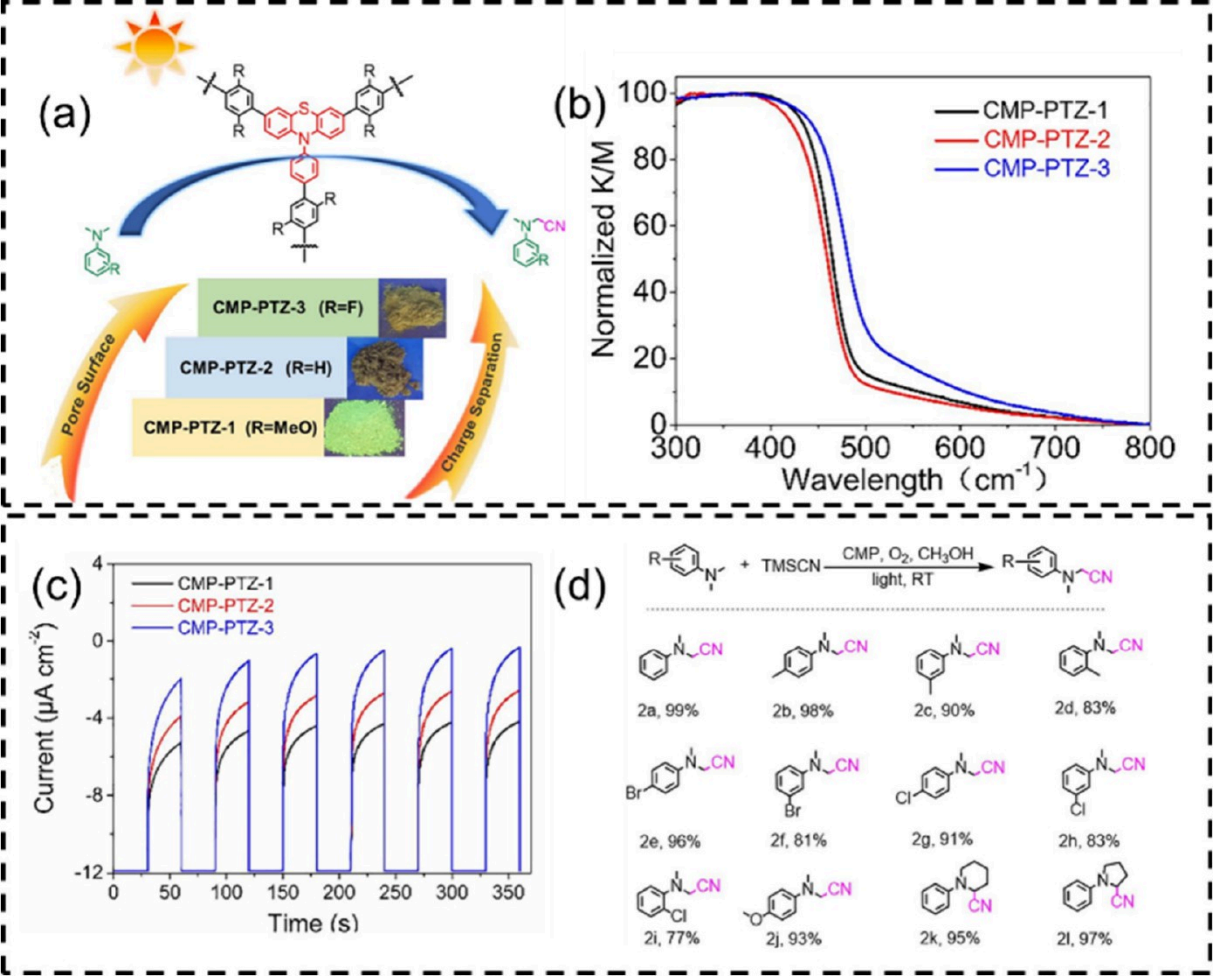
(a) Structures of photoredox-active CMPs; (b) UV–vis diffuse reflectance spectra; (c) photocurrent response; (d) CMP-PTZ-3-catalyzed cyanation of *N*,*N*-dimethylaniline and derivatives. Adapted with permission from ref 39. Copyright 2020 Elsevier.

**Figure 5:**
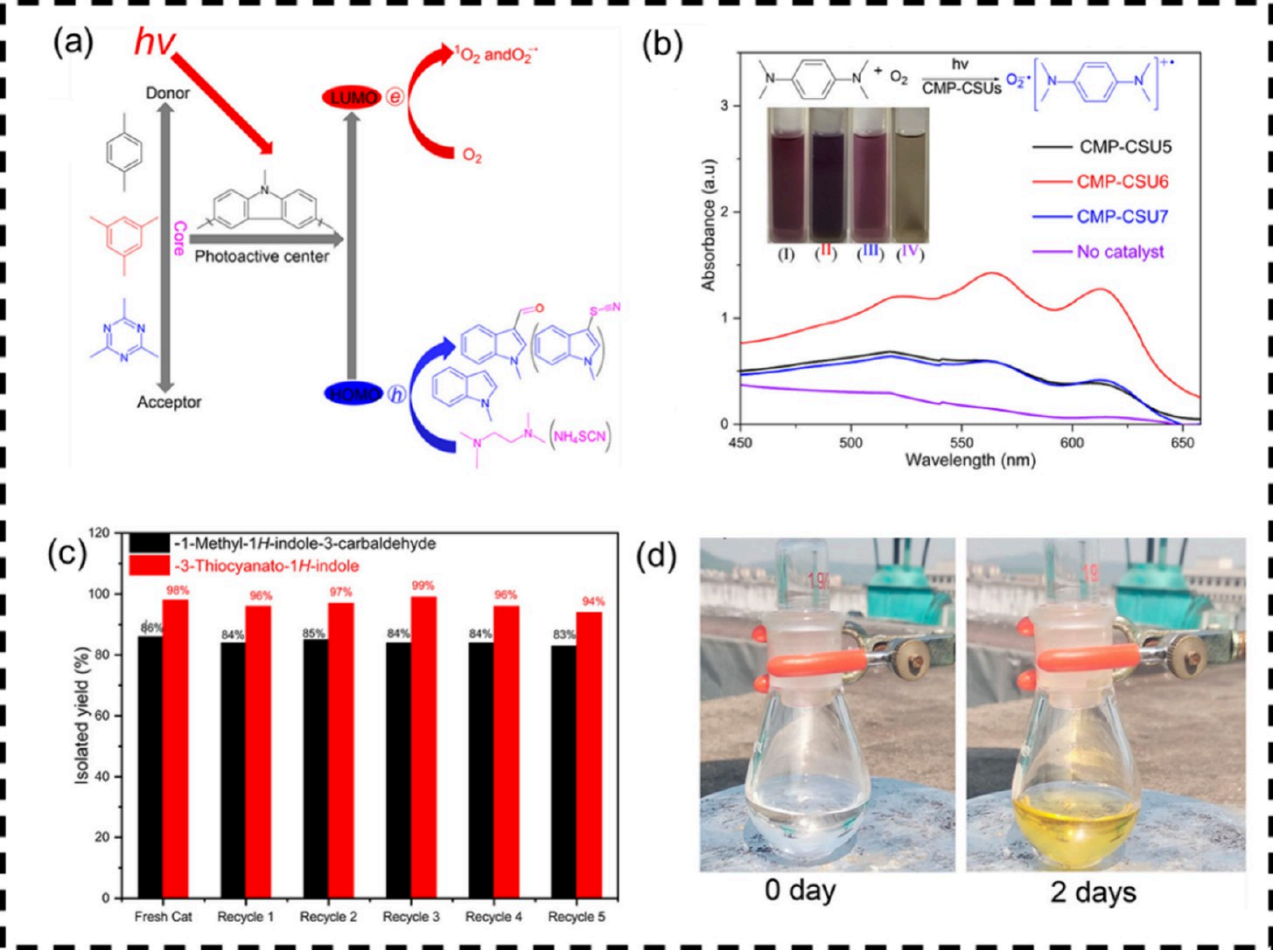
(a) Structures of CMP-CSU*x* (*x* = 5, 6, 7) and their applications in C-3 functionalization of indoles; (b) UV–vis spectra and photographs of TMPD cationic radicals generated by CMP-CSUs under light and oxygen (inset: I, CMP-CSU5; II, CMP-CSU6; III, CMP-CSU7; IV, no catalyst); (c) Recyclability of CMP-CSU6 in C-3 formylation and thiocyanation of indoles; (d) C-3 formylation of 1-methyl-1*H*-indole under solar radiation in Changsha, China (25–28 °C, 08/04/2018–09/04/2018). Adapted with permission from ref 40. Copyright 2018 American Chemical Society.

**Figure 6:**
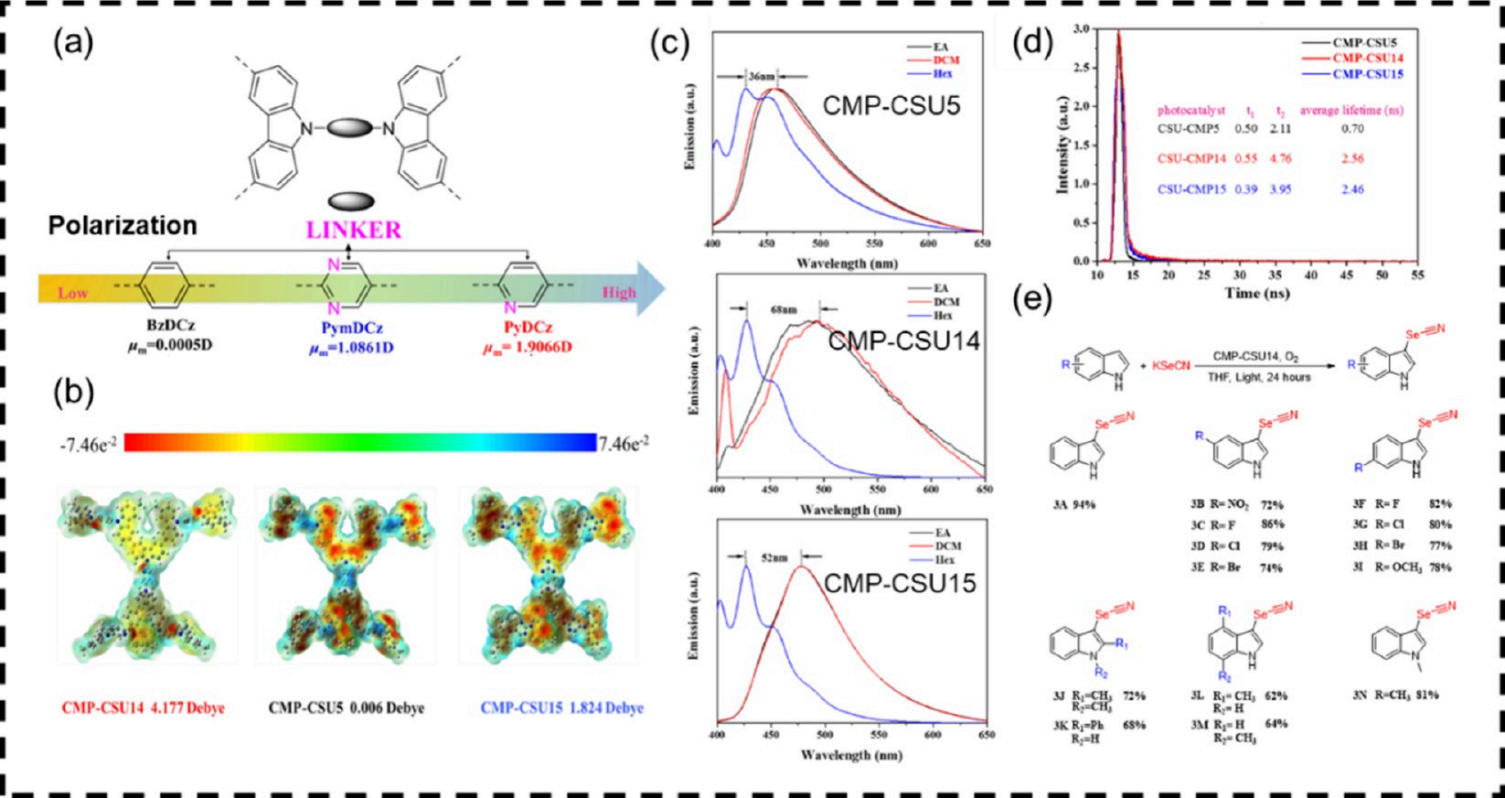
(a) Design strategy of polarized CMP-CSU monomers; (b) molecular dipoles of CSU-CMP oligomers; (c) CMP-CSUs in different polar solvents (0.2 mg sample in 4 mL solvent; EA, ethyl acetate; DCM, dichloromethane; Hex, hexane); (d) photoluminescence decay traces; (e) substrate scope for C-3 selenocyanation of indoles. Adapted with permission from ref 41. Copyright 2021 Royal Society of Chemistry.

### 4.2 Polarization in CPPs

Charge separation and transport efficiency can be pushed forward by a polarization strategy that creates built-in electric fields through large molecular dipoles and improved crystallinity.^41^ Carbazole-based CPPs, known for their strong electron-donating ability, were selected as donor units for generating molecular dipoles (Figure 6). By variation of the electron-accepting units during polymer synthesis, a series of carbazole-based CMP-CSUs (CMP-CSU-5, CMP-CSU-14, and CMP-CSU-15) were constructed, featuring tunable polarization determined by the number of nitrogen atoms in the aromatic modulators.

**Figure 7:**
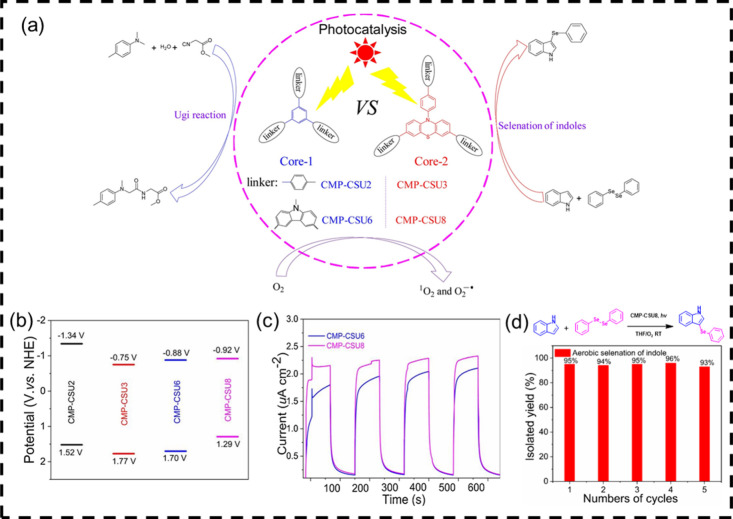
(a) Structures of the photoredox catalysts CMP-CSUs and their applications in photocatalytic Ugi-type reaction and aerobic selenation of indoles; (b) HOMO and LUMO band positions of CMP-CSUs; (c) transient photocurrent response of CMP-CSUs under visible-light irradiation; (d) recyclability tests of CMP-CSU8 in selenation of indole. Adapted with permission from ref 42. Copyright 2021 Elsevier.

Compared with the pristine CMP-CSU-5, the CMP-CSUs incorporating electron-deficient linkers exhibited stronger polarity and, thus, a more pronounced built-in electric field. In the C-3 selenocyanation of indoles, CMP-CSU-14 (pyridine linker, dipole moment 4.177 D) demonstrated higher activity than CMP-CSU-15 (pyrimidine linker, dipole moment 1.824 D), and both outperformed the benzene-linked CMP-CSU-5 (dipole moment 0.006 D). In our view, increasing the molecular dipole moment through acceptor design obviously raises the photocatalytic efficiency.

### 4.3 Extension of the Charge Transfer Pathway

As is well known, mobile charge carriers are supported by the delocalized valence and conduction wave functions formed by the π (bonding) and π* (antibonding) orbitals. Therefore, extending the π-conjugated dimension, like tailoring the polymer chain, surrounding the connection form of the block will also increase the charge separation efficiency.

Polymer chains with extended π-systems can be created using conjugated construction linkers. By adding a π bridge between the donor and acceptor units, D-π-A type conjugated polymer photocatalysts have been made to increase the conjugation degree of the polymer chains and adjust the chemical composition of polymer photocatalysts.^42^ Given these advantages, we reported the rational design and synthesis of a redox-active CPP (CMP-CSU8), using phenothiazine, containing both nitrogen and sulfur heteroatoms and bearing strong electron-donating power, carbazole as the electron donors, and benzene as the π-spacer (Figure 7). In comparison to the phenyl-linked networks CMP-CSU6 (phenothiazine free) and CMP-CSU3 (carbazole free), CMP-CSU8 could serve as an efficient and heterogeneous photocatalyst for organic synthesis, as exemplified by the Ugi reaction and aerobic selenation of indoles. The extended π-conjugated networks of CMP-CSU8 contribute to narrow bandgaps and reduced charge recombination, resulting in good charge-separation efficiency and best photocatalytic activity.

**Figure 8:**
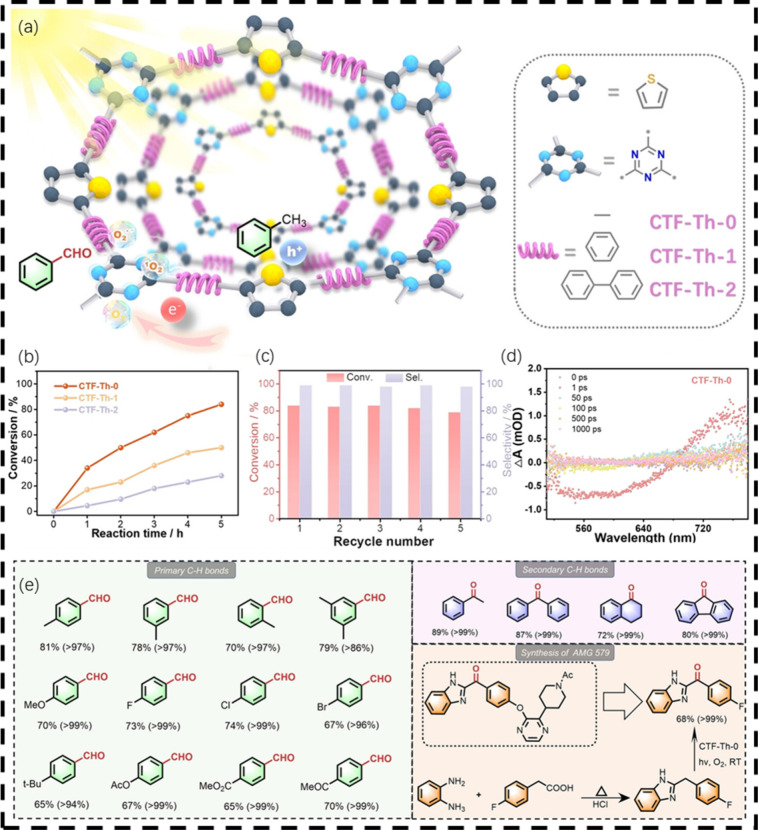
Preparation of CTFs and their photocatalytic performance. (a) Schematic illustration of the molecular engineering strategy of CTFs for the photocatalytic selective oxidation of toluene. (b) Efficiency comparison of the photocatalytic selective oxidation of toluene over different CTFs. (c) Catalytic durability of CTF-Th-0. (d) Transient absorption signals of CTF-Th-0. (e) Substrate scope for the visible-light-induced aerobic oxidation of C–H bonds in hydrocarbons. Adapted with permission from ref 43. Copyright 2024 Wiley-VCH.

Recently, we further developed a system of thiophene-based covalent triazine frameworks (CTF-Th-*x*, *x* = 0,1,2) with tunable length of the benzene linker, which demonstrated exceptional catalytic performance in the selective aerobic oxidation of inert hydrocarbons under mild conditions (Figure 8).^43^ Through systematic variation of the phenyl linker length between the thiophene donor and triazine acceptor units, we achieved precise control over photogenerated hole density, which directly governs photocatalytic efficiency. The optimized CTF-Th-0 configuration, featuring direct thiophene-triazine conjugation, demonstrated exceptional hole accumulation, simultaneously enhancing both charge mobility and the critical C­(sp^3^)–H bond activation step. Combined femtosecond transient absorption (fs-TA) measurements and DFT calculations unambiguously identified the thiophene sulfur sites as the catalytic centers, where photogenerated holes localize to drive substrate oxidation. By precise modulation of the phenyl spacer length between the thiophene and triazine units, we control the formation of photogenerated holes, which subsequently influences the photocatalytic performance. This strategy offers a new approach to metal-free photocatalysts and contributes to further advancements in the field.

**Figure 9:**
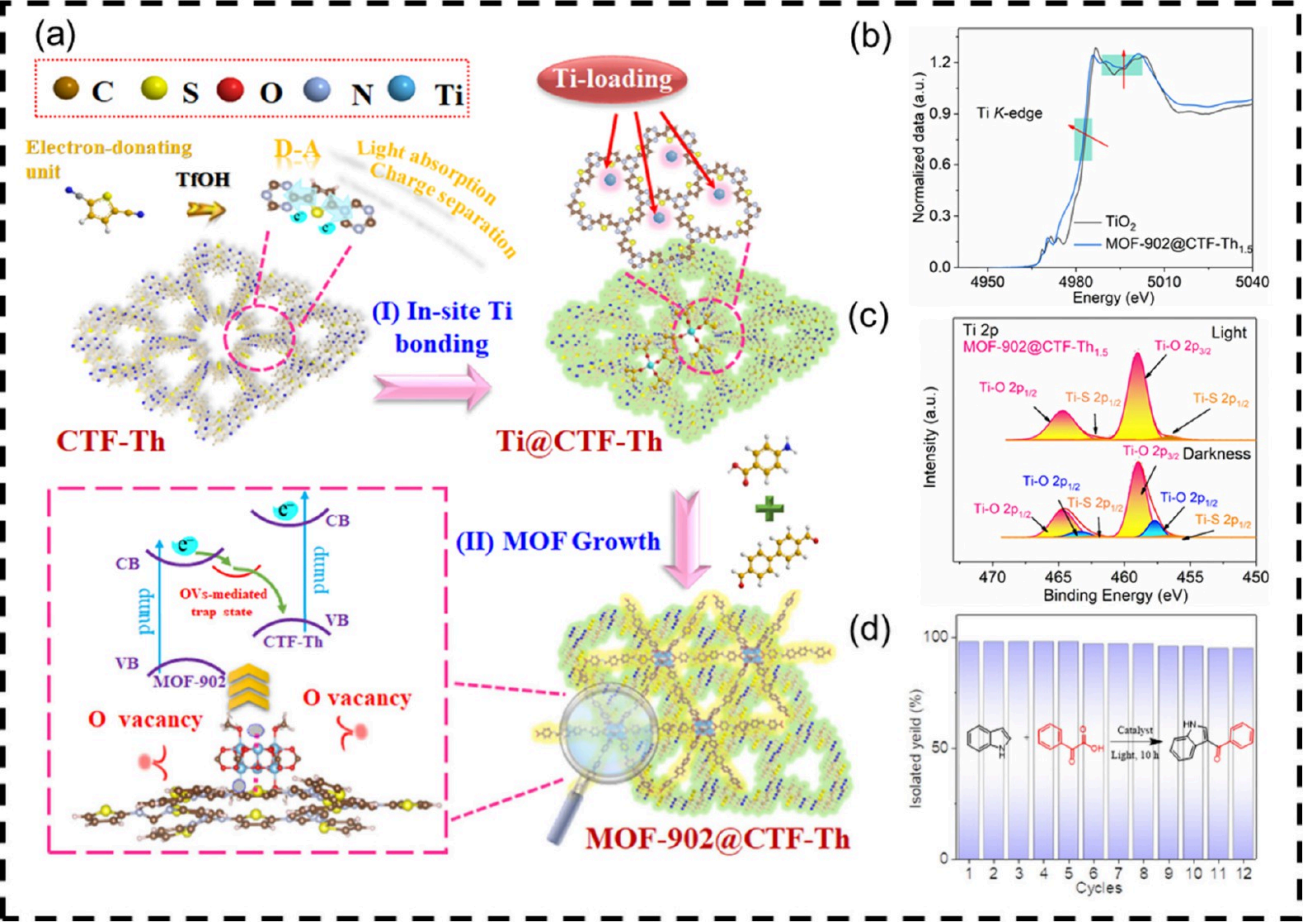
(a) Schematic presentation of the synthetic route of the MOF-902@CTF-Thx heterostructure; (b) XANES spectra of MOF-902@CTF-Th_1.5_; (c) *in situ* irradiated XPS for Ti 2p of MOF-902@CTF-Th_1.5_ in the dark or under 365 nm LED irradiation; (d) cycling stability test of C3-acylation of indoles for MOF-902@CTF-Th_1.5_. Adapted with permission from ref 44. Copyright 2023 Wiley-VCH.

**Figure 10:**
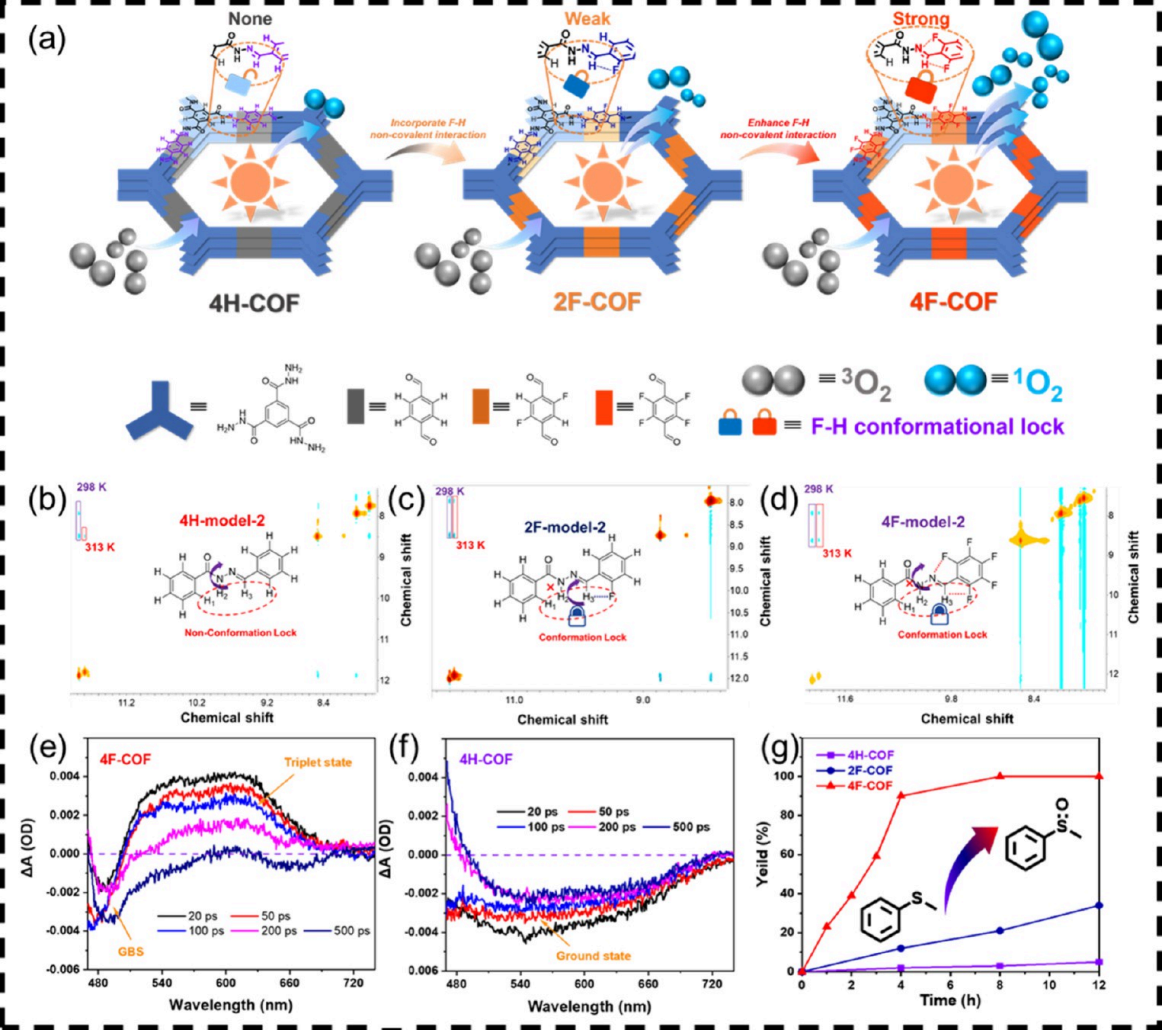
(a) Schematic presentation for the synthesis of COFs and views of the conformational lock in hydrazone-linked COFs for enhanced singlet oxygen generation; 2D NOESY spectra of (b) 4H-model-2, (c) 2F-model-2, and (d) 4F-model-2; (e, f) differential absorption spectra of 4H-COF and 4F-COF at various time delays between 20 and 500 ps after laser excitation at 400 nm; (g) the time course of 4H-thioanisole conversion catalyzed by H-COF and F-COF. Adapted with permission from ref 45. Copyright 2024 Wiley-VCH.

### 4.4 Constructing Heterojunctions

Building heterojunctions within CPPs is capable of improving the charge transfer. By combination of two or more semiconducting materials with complementary band structures, heterojunctions enable more efficient photon absorption and charge separation. In CPPs, forming heterojunctions with other materials, such as semiconductor nanoparticles or metal–organic frameworks (MOFs), can enhance charge-directed transport and, in turn, photocatalytic activity.

Since interfacial charge transfer in heterostructures often is kinetically slow, its modification is challenging. We constructed the heteroatom-induced interface for generating the titanium-organic frameworks (MOF-902)@thiophene-based covalent triazine frameworks (CTF-Th) nanosheets S-scheme heterojunctions with controllable oxygen vacancies (OVs).^44^ Specifically, Ti atoms were first linked onto the heteroatom site of CTF-Th nanosheets before growing into MOF-902 via an interfacial Ti–S bond, resulting in OVs (Figure 9). The enhanced interfacial charge directed transfer generated by moderate OVs in predesigned S-scheme nanosheets was validated using *in* *situ* X-ray photoelectron spectroscopy (XPS), extended X-ray absorption fine structure (EXAFS) spectroscopy, and density functional theory (DFT) calculations. The heterostructures enhanced the efficiency in the photocatalytic C3-acylation of indoles under mild circumstances, yielding 8.2 times more than pure CTF-Th or MOF-902 and allowing for a wider range of substrates (15 instances). Our study introduces an effective S-scheme CTF-based heterostructure photocatalyst and establishes a framework for designing alternative photocatalyst systems for efficient green energy conversion.

## 5 Regulation of Oxygen-Active Intermediates

Precise control of oxygen-active intermediates (e.g., ^•^O_2_^–^, H_2_O_2_, or ^•^OH) is crucial for photocatalytic and electrocatalytic efficiency. While homogeneous catalysts often achieve such regulation through tailored ligand environments, heterogeneous systems typically lack the structural precision required for fine-tuning oxygen-active species. CPPs with their well-defined molecular architectures and tunable functional groups provide an ideal platform for manipulating the formation, stabilization, and reactivity of oxygen-active intermediates. By precisely engineering the chemical microenvironment and electronic structure of active sites, CPPs can regulate the binding affinity, lifetime, and reaction pathways of these key intermediates, thereby optimizing the catalytic selectivity and activity in oxygen-involved reactions.

### 5.1 Conformational Regulation for Controlled Oxygen Species Generation

Given that the microenvironment of active sites is influenced by introduction of noncovalent interactions in a 2D COF, they can regulate their intraconformations and periodic columnar π-arrays on the basis of the elaborate design and synthesis of COF-based materials, leading to amplified photocatalytic performances and stability (Figure 10).^45^ Through intralayer F–H noncovalent interactions, we establish a “conformational lock” (CL) strategy to regulate the photocatalytic activity of a hydrazone-linked 2D COF. When compared to fluorine-free 4H-COF, the fluorinated 2D COFs (4F-COF and 2F-COF) with F–H noncovalent interaction exhibit a constrained conformation. The 2D nuclear Overhauser effect spectroscopy (2D NOESY) measurement presents clear experimental evidence of the high degree of conformational restraint in the fluorinated model compounds, proving the significant role of fluorinated units in the formation of CL. DFT calculations disclose that the larger reduced density gradient (RDG) area unambiguously confirms a stronger F–H noncovalent interaction on the backbone of 4F-COF than that of 4H-COF. By enabling the ISC process, the “CL” effect provides a general route to modulate the photoexcitonic process, enhancing the selective activation of oxygen molecules to singlet oxygen. To demonstrate the efficacy of this approach, typical ^1^O_2_-dependent photocatalytic transformations are used as model reactions, such as the oxidation of thioether, 1,3-diphenylisobenzofuran, and [4 + 2] cycloaddition of 1,3-dienes. More significantly, the “CL” effect enhances the stability of COFs, preserving porosity, crystallinity, and photocatalytic activity even after 7 cycles. This work demonstrates that atomic-scale modulation of intralayer noncovalent interactions provides a novel strategy for regulating oxygen-active species generation in 2D-COFs.

### 5.2 Hydroxyl Radical Mediated Oxidation Reaction

Many photocatalytic studies have reported precise control over the production of oxygen-active intermediates, and most focus on accelerating substrate reactions to yield desirable end products. However, the influence of the production of oxygen-active species on the chemical microenvironment of catalytic sites has seldom been investigated and remains largely elusive.

With this in mind, an organic photocatalytic system using covalent triazine/heptazine-based frameworks (CTF-TB/CHF-TB) has been constructed and served as a regulator to “chelate” and decompose the *in situ* generated H_2_O_2_ into hydroxyl radicals (**·**OH). By altering the building block with strong-electron-deficient and rich-nitrogen characteristics of CPPs, the heptazine sites can be easily regulated to modulate the adsorption and activation of H_2_O_2_ (Figure 11).^46^ The substrate adsorption energy of CPPs can be influenced, following the order CTF-TB < CHF-TB based on the theoretical calculation. Moreover, the incorporation of heptazine units enhances the charge separation efficiency of the photocatalysts, and its bipyridine-like motif strengthens the decomposition of H_2_O_2_. The **·**OH generating route is optimized by these features, and **·**OH acts as a C–H cleavage initiator in the B–V oxidation process, taking the place of metal activation in previously documented catalytic materials to produce the following sequence: O_2_ → H_2_O_2_ → **·**OH → Ph–CO**·** → Ph–COOO**·**. Under milder circumstances, it finally interacts with the substrate cyclohexanone and produces ϵ-caprolactone in high yield. Our study creates a novel technique to mildly generate ϵ-caprolactone using nonmetallic organocatalysts under visible light.

**Figure 11:**
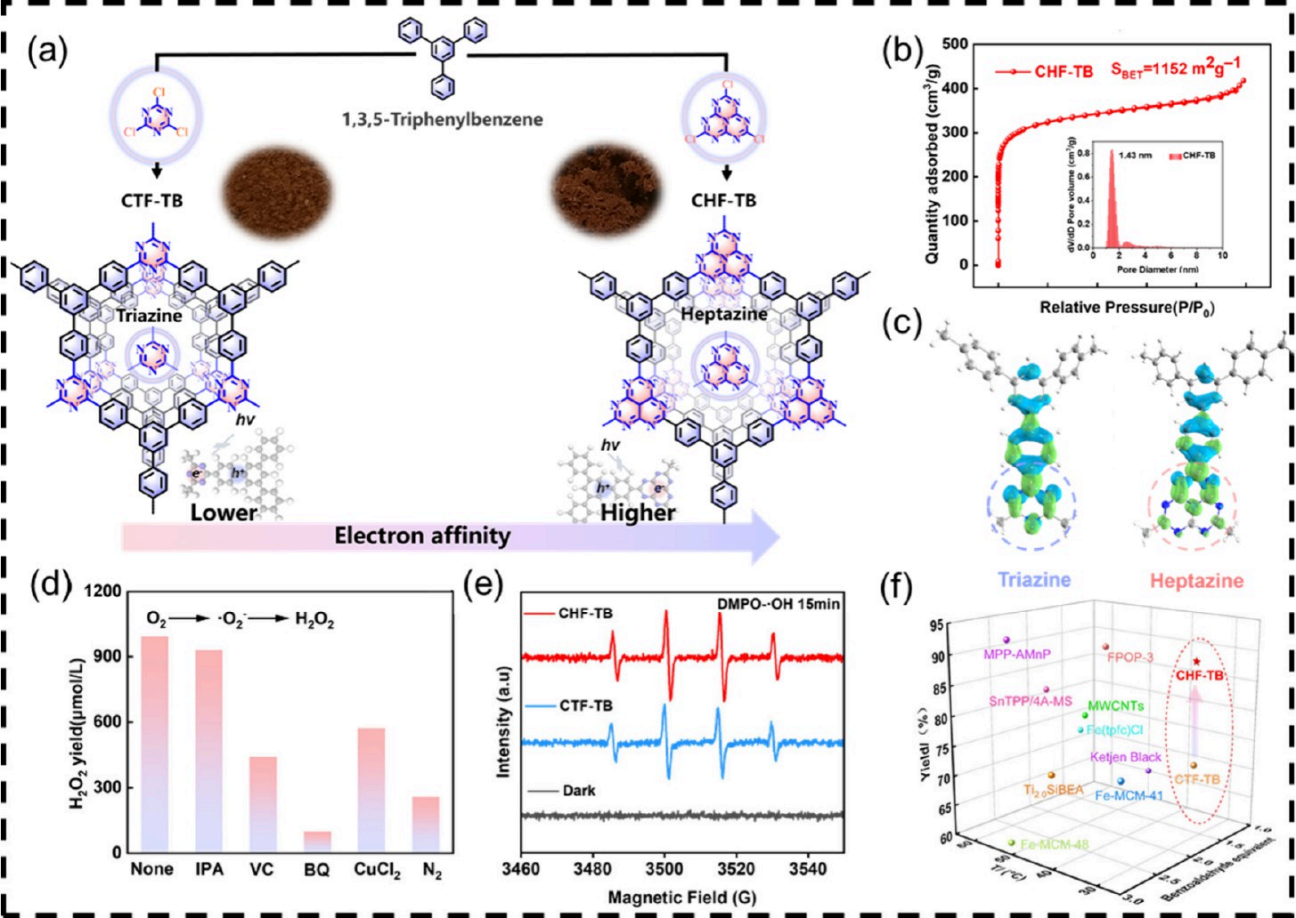
(a) The design principle for the synthesis of CHF-TB/CTF-TB; (b) nitrogen sorption isotherms at 77 K. Inset: pore size distribution plots; (c) electron–hole distribution of typical fragments in CHF-TB (right) and CTF-TB (left) in the excited states; (d) photocatalytic H_2_O_2_ production experiments with the addition of different sacrificial agents (1 mM); (e) EPR spectra of DMPO-·OH; (f) summary of the B-V oxidation of cyclohexanone with O_2_ catalyzed by various catalysts. Adapted with permission from ref 46. Copyright 2024 Wiley-VCH.

## 6 Conclusions and Perspectives

This Account summarizes our recent contributions to unveil the structure–property relationships of photocatalytic organic synthesis in triazine- or carbazole-based CPPs. We share our experience of how the molecular structure can be tailored to perform CPP-based photocatalysis better. Above all, by engineering molecular units, bridging bonds, and functional groups of CPPs, light harvesting can be finely programmed, and molecular affinity, recognition, and adsorption can be achieved during photocatalysis. Second, altering the donor–acceptor systems, π-bridging units, and the interfacial defects of heterostructure in CPPs can change the facial catalytic sites to modulate kinetics in directional charge transfer in photocatalysis. Third, oxygen-active species can be tailor-made by precisely crafting the microenvironment and engineering conformational dynamics of catalytic active sites. Accordingly, the intrinsic activity and selectivity of catalytic sites will be further raised. To note, the synergy of the above factors, such as simultaneous regulation of directional charge transfer and oxygen-active intermediates, has been successfully realized to afford satisfied photoactivity and selectivity in CPP-based catalysis.

Despite the progress made here, the development of orientation-enhanced charge transport and precise regulation of oxygen-active intermediates in heterogeneous photocatalysis remains in a nascent stage. This is practically the starting point for oxygen-active intermediate-modulated catalysis over CPP-based materials with many challenges remaining. First, at present, it is imperative to come up with more strategies for oxygen-active intermediate modulation in CPP-based catalysts. More factors, including triplet exciton concentration and various intermolecular interactions (electrostatic, van der Waals, and coordination interactions) could be strategically incorporated to facilitate substrate enrichment, molecular organization, and intermediate stabilization. Meanwhile, the pore space partition, a unique strategy that can tune pore geometric features and strengthen the stability of CPPs, might also be promising to handle the oxygen-active intermediates. Second, in addition to accurate control over the single aspect of the oxygen-active intermediates, simultaneous regulation of the directional mass/electron transfer and intrinsic behavior of catalytic sites would be better in mimicking the complex enzymatic catalysis for high performance. Thus, the detailed mechanisms regarding the specific interaction between catalytic sites and intermediates in CPP-based materials await in-depth studies. Not only the interaction but also the reaction transition states and the well-defined and tailorable CPP structures should be of great support in regulation and understanding. Based on this ideal platform, more advanced characterizations and *operando* techniques might be necessary to obtain insightful information.

On the basis of these advances, we are motivated to share our vision on critical directions warranting prioritized investigation.

(1) Expanded photoelectric property and intermediate engineering strategies. Current methodologies must evolve beyond basic structural modifications to incorporate multivariate crystalline engineering. Systematic integration of secondary interactions, including hydrogen-bonding networks, electrostatic gradients, and coordination motifs, could enable substrate preorganization and transition-state stabilization akin to enzymatic pockets. The emerging concept of pore-space partition, which permits simultaneous optimization of pore geometry and framework stability, offers a promising avenue for creating hierarchical catalytic environments with tailored substrate diffusion pathways and confined reactive volumes.
(2) Pore confinement engineering. In the field of photocatalytic organic conversion, the pore confinement effect offers multifaceted strategies for enhancing catalytic performance. By restricting the reaction space of active sites, pore structures can modulate the surface electronic properties of catalysts and optimize reactant–catalyst interactions, thereby significantly improving reaction rates and selectivity. Furthermore, pore confinement helps stabilize intermediates and prevents the aggregation of active sites. Throughout the photocatalytic process, functionalized pore design, such as the incorporation of transition metals, creation of defect sites, enhancement of intrinsic catalytic activity, construction of cross-linked frameworks, and optimization of ion transport channels, can synergistically promote the separation of photogenerated charges, enhance reaction kinetics, and improve contact efficiency between substrates and active sites.
(3) Synergistic regulation of multiscale processes. Mimicking biological catalysis of high complexity and efficiency requires concurrent control over electronic structures and nanoconfined reaction fields. Developing synergistic regulation protocols that couple directional charge transport with catalytic site-driven intermediate activation could bridge the performance gap between artificial and natural catalytic systems. Such dual optimization must address the kinetic coupling between photogenerated carrier dynamics and localized intermediate concentration gradients.
(4) Mechanistic elucidation through high-end characterization tools. The atomistic interplay between CPP architectures and catalytic outcomes demands rigorous interrogation using multimodal approaches. Time-resolved spectroscopic techniques (e.g., transient absorption spectroscopy) should be combined with *operando* X-ray absorption fine structure (XAFS) analysis to map real-time evolution of active sites during photocatalysis. Computational modeling of host–guest complexes, especially transition-state configurations confined within porous architectures, could elucidate the mechanistic interplay between charge-directed transport dynamics and oxygen-active species regulation in governing reaction selectivity. Establishing structure–activity descriptors through machine learning-assisted analysis of crystalline parameters (e.g., interlayer distances and pore structure) may accelerate catalyst design. This emerging paradigm nevertheless faces critical challenges that demand systematic efforts. The development of multivariate synthetic methodologies, coupled with characterization platforms, will be essential for transforming CPP-based systems from model catalysts into practical artificial photosynthetic devices.
(5) Emerging high-value-added organic synthesis. Although significant progress has been made in covering major categories of organic reactions, opportunities remain for designing photocatalytic systems that combine high efficiency and selectivity, particularly for industrially relevant processes. For example, the synthesis of bulk chemicals such as cyclohexanone oxime, currently produced under energy-intensive redox conditions, could be greatly improved by employing light-driven catalysis combined with confined nanochannels, enabling milder reaction conditions and greener synthetic pathways. Furthermore, chiral photocatalysis based on CPPs constitutes an emerging and essential branch of research. Advances in the deliberate construction of chiral pore environments or asymmetric active sites within CPPs, along with the expansion of enantioselective reaction types, will open new avenues for sustainable asymmetric synthesis. These developments will not only broaden the scope of photocatalytic organic conversions but also promote the adoption of solar-driven technologies in the production of fine chemicals and pharmaceuticals.

While this research topic is widely unexplored, the systematic elucidation of both advancements and persistent challenges presented in this Account serves to strengthen the theoretical foundations for biomimetic photocatalyst design. Our findings regarding charge-directed transport and oxygen-active species regulation in CPP-based systems have revealed critical structure–activity relationships, particularly in photoinduced charge separation efficiency and task-specific catalytic sites. These mechanistic insights, derived from our experimental investigations into donor–acceptor configurations, tautomerism, and interfacial host–guest interactions, establish a paradigm for rational design of heterogeneous photocatalytic architectures. By elucidating the synergistic interplay between charge-directed transport modulation and oxygen-active species regulation, we demonstrate viable pathways for enhancing quantum yields in artificial photosynthetic systems. The fundamental principles outlined herein not only advance our current understanding of photocatalysis but also pave the way for developing next-generation energy conversion platforms through the accurate management of photophysical processes and interfacial reaction environments.
